# Large-Scale Identification of Known and Novel RRNPP Quorum-Sensing Systems by RRNPP_Detector Captures Novel Features of Bacterial, Plasmidic, and Viral Coevolution

**DOI:** 10.1093/molbev/msad062

**Published:** 2023-03-17

**Authors:** Charles Bernard, Yanyan Li, Philippe Lopez, Eric Bapteste

**Affiliations:** Institut de Systématique, Évolution, Biodiversité (ISYEB), Sorbonne Université, CNRS, Muséum National d'Histoire Naturelle, Paris, France; Unité Molécules de Communication et Adaptation des Micro-organismes (MCAM), CNRS, Muséum National d’Histoire Naturelle, Paris, France; Department of Computational Biology, University of Lausanne, Genopode, Lausanne, Switzerland; Unité Molécules de Communication et Adaptation des Micro-organismes (MCAM), CNRS, Muséum National d’Histoire Naturelle, Paris, France; Institut de Systématique, Évolution, Biodiversité (ISYEB), Sorbonne Université, CNRS, Muséum National d'Histoire Naturelle, Paris, France; Institut de Systématique, Évolution, Biodiversité (ISYEB), Sorbonne Université, CNRS, Muséum National d'Histoire Naturelle, Paris, France

**Keywords:** RRNPP, Quorum Sensing, Microbial Communication, Bacteriophages, Plasmids, Host-Virus co-evolution

## Abstract

Gram-positive *Firmicutes* bacteria and their mobile genetic elements (plasmids and bacteriophages) encode peptide-based quorum-sensing systems (QSSs) that orchestrate behavioral transitions as a function of population densities. In their simplest form, termed “RRNPP”, these QSSs are composed of two adjacent genes: a communication propeptide and its cognate intracellular receptor. RRNPP QSSs notably regulate social/competitive behaviors such as virulence or biofilm formation in bacteria, conjugation in plasmids, or lysogeny in temperate bacteriophages. However, the genetic diversity and the prevalence of these communication systems, together with the breadth of behaviors they control, remain largely underappreciated. To better assess the impact of density dependency on microbial community dynamics and evolution, we developed the RRNPP_detector software, which predicts known and novel RRNPP QSSs in chromosomes, plasmids, and bacteriophages of *Firmicutes*. Applying RRNPP_detector against available complete genomes of viruses and *Firmicutes*, we identified a rich repertoire of RRNPP QSSs from 11 already known subfamilies and 21 novel high-confidence candidate subfamilies distributed across a vast diversity of taxa. The analysis of high-confidence RRNPP subfamilies notably revealed 14 subfamilies shared between chromosomes/plasmids/phages, 181 plasmids and 82 phages encoding multiple communication systems, phage-encoded QSSs predicted to dynamically modulate bacterial behaviors, and 196 candidate biosynthetic gene clusters under density-dependent regulation. Overall, our work enhances the field of quorum-sensing research and reveals novel insights into the coevolution of gram-positive bacteria and their mobile genetic elements.

## Introduction

Quorum sensing is the mechanism by which microbial entities sense when their population density reaches a threshold level and thereupon typically switch from individual to group behaviors (Mukherjee and Bassler 2019). The population density is reflected by the extracellular concentration of a communication signal, produced and secreted by individual entities. The quorum is met when this signal reaches a threshold concentration, at which it starts to be robustly detected and transduced population-wide by its cognate receptor module. If quorum sensing seems to be used by diverse prokaryotic and unicellular eukaryotic lineages (Hornby et al. 2001; Paggi et al. 2003; Sun et al. 2004; Sharif et al. 2008; Tian et al. 2018), most of the knowledge about this communication mechanism comes from the three *Pseudomonadota* (formerly *Proteobacteria*), *Actinomycetota* (formerly *Actinobacteria*), and *Bacillota* (formerly *Firmicutes*) bacterial phyla. In the *Pseudomonadota*/*Proteobacteria* and *Actinomycetota/Actinobacteria* phyla, the communication signals typically are small molecules synthesized by enzymes (Papenfort and Bassler 2016; Polkade et al. 2016), whereas in the *Bacillota/Firmicutes* phylum, these are oligopeptides, matured from genetically encoded propeptides (Bhatt 2019). Peptide-based quorum-sensing systems (QSSs) can be divided into two main categories: those with a receptor module composed of a membrane-bound sensor coupled with an intracellular response regulator (two-component system) like the ComX-ComQ-ComP-ComA of *Bacillus subtilis* (Sturme et al. 2002) and those in which the receptor is an intracellular transcription factor (or a protein inhibitor) that gets either turned-on or -off upon binding with the imported communication peptide (one-component system) (Rocha-Estrada et al. 2010; Neiditch et al. 2017). The latter are generally included under the term RRNPP, named after the five first experimentally characterized subfamilies of such receptors: Rap (*Bacillus* genus), Rgg (*Streptococcus* genus), NprR (*Bacillus cereus* group), PlcR (*B. cereus* group), and PrgX (pCF10 plasmid of *Enterococcus faecalis*) (Do and Kumaraswami 2016; Perez-Pascual et al. 2016; Neiditch et al. 2017).

The initial members of the RRNPP group of QSSs were reported to trigger key biological pathways when their encoding population reaches high densities: from virulence (Rgg, PlcR) to competence (Rgg, Rap), necrotropism (NprR), sporulation, biofilm formation (Rap, NprR), and inhibition of conjugation (PrgX) (Do and Kumaraswami 2016; Perez-Pascual et al. 2016; Neiditch et al. 2017). Considering that the virulence of *Bacillus* and *Streptococcus* pathogens may cause infectious diseases in humans (Baldwin 2020; Lannes-Costa et al. 2021), that the spore is the transmissive form of many *Bacillus* and *Clostridium* human pathogens (Mallozzi et al. 2010), that biofilms contribute to infections or food poisoning (Costerton et al. 1999; Høiby et al. 2011; Galié et al. 2018), and that competence and conjugation are responsible for the spread of antibiotic resistance genes (von Wintersdorff et al. 2016), RRNPP QSSs are directly linked to central health issues.

Interestingly, the case of the plasmidic PrgX system illustrates that RRNPP QSSs may be not only present on bacterial chromosomes but also on mobile genetic elements (MGEs). However, conjugative elements are not the only MGEs relying on RRNPP QSSs as a means to assess their population density. Indeed, in 2017, Erez et al. made the groundbreaking discovery of the viral “arbitrium” communication system, an RRNPP QSS encoded by temperate phages of *Bacillus* and guiding the lysis–lysogeny decision upon *Bacillus* infection (Erez et al. 2017; Stokar-Avihail et al. 2019).

**Fig. 1.**
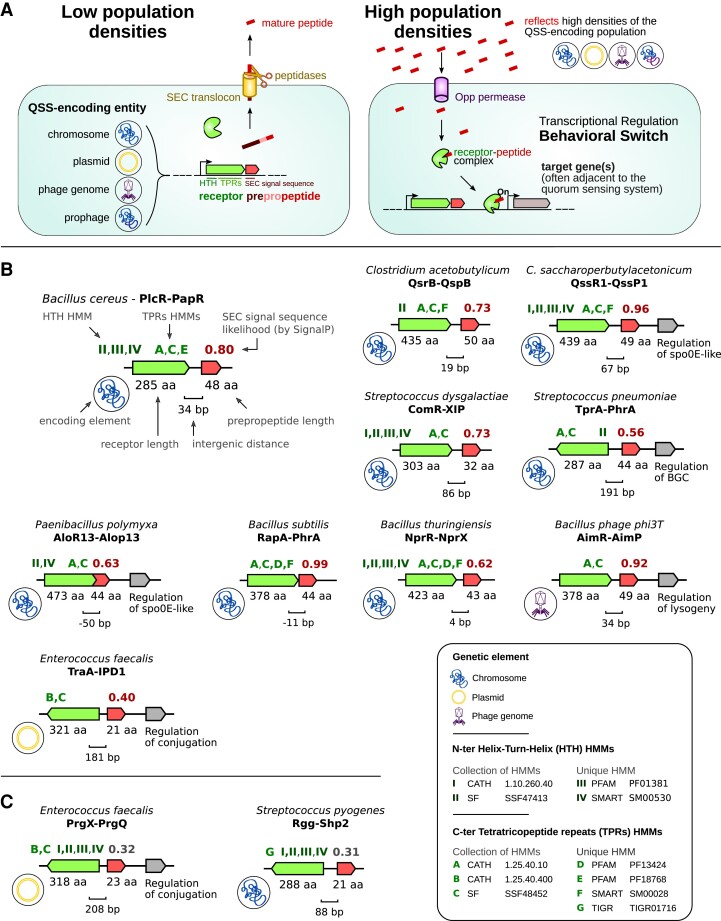
Characteristics of RRNPP QSSs. (*A*) Canonical molecular mechanism of communication via an RRNPP QSS. . An RRNPP QSS can be encoded by chromosomes, plasmids, phage genomes, or prophages (phage genomes inserted within the bacterial genome). Either way, upon bacterial expression, the propeptide is secreted via the bacterial SEC translocon and is cleaved extracellularly into a short mature communication peptide. As a QSS-encoding element replicates, the communication peptide accumulates in the extracellular environment. At high concentrations of the peptide, reflecting a quorum of bacterial cells, plasmids, and/or (pro)phages, the peptide starts to be frequently imported within bacterial cells. In bacterial cells hosting the QSS-encoding genetic element(s), the communication peptide binds to the TPRs of its cognate cytosolic receptor. Consequently, the receptor gets either turned-on or -off as a protein inhibitor or as a transcription factor, which is at the basis of density-dependent regulations of target proteins or genes. As a result, a behavioral transition is initiated at the scale of the entire QSS-encoding population. (*B*) Common features between experimentally validated RRNPP QSSs. Each genomic context corresponds to the representative QSS of an experimentally validated subfamily of RRNPP QSSs. . A gray gene indicates an adjacent target gene (or set of genes) demonstrated to be regulated by the QSS. The legend on the top-left corresponding to the PlcR-PapR QSS indicates all genomic features being displayed for each QSS. The different QSSs share a computationally testable signature of five criteria: 1) the propeptide is small; 2) the propeptide is secreted by the SEC translocon (computationally testable by SignalP); 3) the receptor is ∼250–500aa long; 4) the receptor harbors TPRs involved in the recognition of the mature communication peptide (computationally testable by HMMs of TPRs); and 5) the receptor and the propeptide genes are direct neighbors. (*C*) RRNPP QSSs involving a secretion of the propeptide via the alternative PptAB translocon. Consistently, SignalP did not predict a SEC-dependent secretion for them (as shown by a SEC-secretion likelihood score colored in gray).

In total, to our knowledge, we can count today 11 subfamilies of RRNPP receptors with experimental evidence of interaction with a communication peptide: the five aforementioned initial RRNPP members Rgg, Rap, NprR, PrgX, and PlcR (which can be divided into the PlcR and TprA subfamilies [Hoover et al. 2015]) (Neiditch et al. 2017) and the six following additional members:

- TraA—plasmids of *E. faecalis* (Kohler et al. 2019),
- AimR—temperate phages of *Bacillus* (Stokar-Avihail et al. 2019),
- ComR—*Streptococcus* genus (Shanker et al. 2016),
- AloR—*Paenibacillaceae* family (Voichek et al. 2020),
- Qsr—*Clostridium acetobutylicum* (Kotte et al. 2020), and
- QssR—*Clostridium saccharoperbutylacetonicum* (Feng et al. 2020) (fig. 1*B*)

Yet, the genetic diversity of RRNPP QSSs may not have been fully explored, as hinted, for instance, by the candidate receptors of *E. faecalis* reported to harbor local similarities with regions of Rap, PlcR, or Rgg (Parthasarathy et al. 2020). Hence, new communication codes as well as novel density-dependent evolutionary strategies likely await to be discovered. These discoveries not only could transform our views of microbial interaction, adaptation, and evolution, but also could have major practical outcomes as novel communication systems could regulate the production of new antimicrobial compounds (Hoover et al. 2015; Rued et al. 2021) or could underlie adaptive mechanisms by which some human pathogens acquire virulence (Edwards et al. 2016, 2019; Do et al. 2017, 2019). However, expanding this diversity requires overcoming an important challenge: identifying candidate systems beyond close homologs of already known RRNPP subfamilies.

Conveniently, we noticed that the members of all the aforementioned experimentally validated RRNPP subfamilies share a common signature of five criteria (fig. 1*B*): 1) the propeptide is a small protein (10–100aa); 2) the propeptide is secreted via the SEC translocon and further matured by exopeptidases into a communication peptide (with the exception of propeptides of short hydrophobic peptides (SHPs) and PrgQ mature peptides associated with Rgg and PrgX receptors that are translocated via the PptAB export system[Neiditch et al. 2017]); 3) the receptor has a length comprised between 250 and 500aa; 4) the receptor harbors tetratricopeptide repeats (TPRs), which are structural motifs involved in the binding of small peptides (in this case, the cognate communication peptide); and 5) the genes encoding the propeptide and the receptor are directly adjacent to each other. Advantageously, a large amount of reference hidden Markov models (HMMs) from the Cath-Gene3D (Sillitoe et al. 2021), Superfamily (Wilson et al. 2009), SMART (Letunic et al. 2021), Pfam (Mistry et al. 2021),⁠ and TIGRFAM (Haft et al. 2013) databases are already available to detect TPRs in protein sequences. Moreover, a tool called SignalP specifically computes the likelihood that proteins harbor a signal sequence for the SEC translocon (Almagro Armenteros et al. 2019) (fig. 1). Consequently, the generic, yet specific signature of RRNPP QSSs could be detectable in silico, without requiring homology searches that would limit the output to representatives of already known QSSs.

**Fig. 2.**
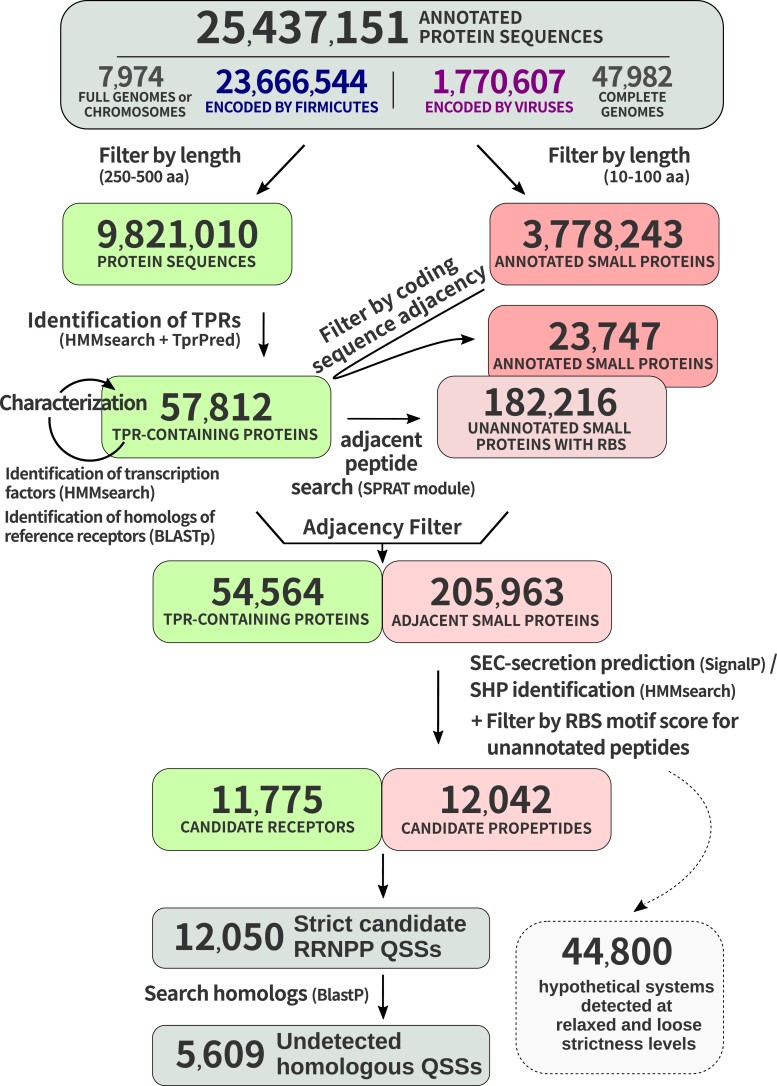
Workflow of RRNPP_detector illustrated with real data from complete genomes/chromosomes of *Firmicutes* and viruses. RRNPP_detector defines candidate RRNPP-type QSSs with a “strict” detection strictness level as tandems of adjacent genes encoding a candidate receptor (250–500aa protein matching HMMs of peptide-binding TPRs) and a candidate propeptide (10–100aa protein predicted by SignalP to be secreted via the SEC translocon or matching the HMM of SHP propeptides). Each green and red rectangle represents a step toward the final identification of “strict” candidate receptors and candidate propeptides, respectively (details in Materials and Methods). The final “strict” receptors and propeptides subsequently serve as queries in a Blastp search to identify additional homologous QSSs that did not pass the conservative thresholds of RRNPP_detector. Additional pairs are predicted with either a “relaxed” or a “loose” detection strictness level (Materials and Methods).

On this basis, we have developed RRNPP_detector, a Python software dedicated to the detection of the RRNPP signature in chromosomes, plasmids, and bacteriophages of gram-positive bacteria, available at https://github.com/TeamAIRE/RRNPP_detector. The fact that the Rgg and PrgX subfamilies involve a secretion of their cognate SHP and PrgQ propeptides via the PptAB translocon rather than via the SEC translocon (Neiditch et al. 2017) implies that some functional RRNPP QSSs can slightly deviate from the previously described canonical signature. Accordingly, RRNPP_detector was designed to identify putative QSSs with three different strictness levels: 1) the “strict” level outputs all candidate receptor–propeptide pairs with the propeptide being annotated or preceded by a high-confidence ribosomal binding site (RBS) motif and either matching the HMM profile of SHPs or predicted to undergo a SEC/SPI-dependent secretion according to SignalP; 2) the “relaxed” level outputs all remaining receptor–propeptide pairs in which the propeptide harbors any of the SP(Sec/SPI), TAT(Tat/SPI), or LIPO(Sec/SPII) secretion tag according to PrediSi (Hiller et al. 2004) or SignalP (Almagro Armenteros et al. 2019); and 3) the “loose” level outputs remaining TPR-containing putative receptors only if found adjacent to a peptide without a detected secretion tag but with a high-confidence upstream Shine–Dalgarno RBS (SD RBS) motif indicative of a likely translation (Shine and Dalgarno 1975; Omotajo et al. 2015), with the cognate peptide being chosen as the most likely translated small protein in the close genomic vicinity of the candidate receptor (fig. 2). Of course, the relaxed and loose outputs are associated with a higher risk of false positives but are nonetheless interesting for exploratory purposes.

To assess the extent of the impact of RRNPP QSSs on microbial community dynamics and evolution, we applied RRNPP_detector against all complete genomes and chromosomes of *Firmicutes* and viruses available on the NCBI. We report a wide phylogenetic, genetic, and functional diversity of RRNPP QSSs that enhances our current knowledge of the coevolution of gram-positive Bacillota and their MGEs.

## Results

### RRNPP_Detector Operates with a Mean Precision of 99% and a Mean Recall of 94% on a Benchmarking Data Set of Genomes with Well-Characterized Repertoires of RRNPP QSSs

Before using RRNPP_detector to identify novel candidate genetic subfamilies of RRNPP QSSs, we wanted to ensure that this software is conservative enough to detect only RRNPP QSSs while being sensitive enough to not miss a substantial number of these QSSs within genomes. Accordingly, we built a benchmarking data set comprising nine genomes in which the repertoire of RRNPP QSSs has been extensively studied to test our method (table 1). These nine reference genomes were chosen to cover a substantial taxonomic diversity and to encode QSSs representative of the diversity of already known RRNPP subfamilies. Of the 50 receptor–adjacent propeptide pairs previously described in the literature for these reference genomes, the “strict” detection module of RRNPP_detector was able to detect 44 pairs and made only 45 predictions (table 1; see Materials and Methods). The averages of recalls and precisions computed for each of the nine genomes were 94% and 99%, respectively (table 1). In particular, the mean precision of 99% offers guarantee that the novel genetic systems that RRNPP_detector was designed to predict could be considered as reliable candidate RRNPP QSSs.

**Table 1:** RRNPP_detector benchmarking results..

Genome	NCBI assembly or genomic accession	Reference	(Predicted vs described) nondegenerated RRNPP-type adjacent pairs											With RBS filter of unannotated peptide			Without RBS filter		
			Rap–Phr	AimR–AimP	PlcR-PapR	TprA-PhrA	NprR–NprX	AloR–AloP	Rgg-Shp	RrgX-PrgQ	TraA-Ipd1	Qsr-Qsp	QssR-QssP	Total predictions	Recall (%)	Precision (%)	Total predictions	Recall (%)	Precision (%)
*Bacillus subtilis* 168	GCA_000009045.1	Even-tov et al. (2016a), Stokar-Avihail et al. (2019)	7/7	1/1										8	100.00	100.00	9	100.00	88.90
*Bacillus cereus* NC7401	GCA_002220285.1	Even-tov et al. (2016b), Stokar-Avihail et al. (2019)	4/4	3/3	1/1		1/1							9	100.00	100.00	9	100.00	100.00
*Bacillus* phage phi3T	GCA_002601445.1	Stokar-Avihail et al. (2019), Bernard et al. (2020)	1/1	1/1										2	100.00	100.00	2	100.00	100.00
*Paenibacillus polymyxa* ATCC 842	GCA_000217775.1	Voichek et al. (2021)						9/13						10	69.23	90.00	12	87.50	83.30
*Streptococcus pyogenes* M1 GAS	GCA_000006785.2	Aggarwal et al. (2014)							2/2					2	100.00	100.00	2	100.00	100.00
*Streptococcus pneumoniae* D39V	GCA_003003495.1	Hoover et al. (2015)				1/1			1/1					2	100.00	100.00	2	100.00	100.00
*Enterococcus faecalis* plasmid pPD1	KT290268.1	Nakayama et al. (1994)									1/1			1	100.00	100.00	1	100.00	100.00
*Clostridium acetobutylicum* ATCC 824	GCA_000008765.1	Kotte et al. (2020)										6/8		6	75.00	100.00	7	85.70	85.70
*Clostridium saccharoperbutylacetonicum* N1–4	GCA_000340885.1	Kotte et al. (2020), Feng et al. (2020)											5/5	5	100.00	100.00	5	100.00	100.00
		Mean performance:													93.80	98.89		97.02	95.32

### RRNPP_Detector Identifies Tens of Thousands of Candidate RRNPP QSSs in Complete Bacterial Chromosomes, Plasmids, and Phage Genomes

As a first step, we launched RRNPP_detector (see Materials and Methods) against the 7,974 complete genomes and chromosomes of *Firmicutes* and the 47,982 complete genomes of viruses available on the NCBI Assembly Database (fig. 2). We describe how such analyses can be easily done with the practical example of viruses in the readme file of RRNPP_detector: https://github.com/TeamAIRE/RRNPP_detector/readme.md. We report the identification of 12,050 pairs predicted with the “strict” mode, which allowed to capture 5,609 additional undetected pairs with the homology search module of RRNPP_detector (see Materials and Methods). Finally, we report 44,800 additional pairs predicted either with the “relaxed” or “loose” modes of detection.

The 12,050 “strict” pairs are distributed in 511 different species, whereas the 48,800 pairs corresponding to the “relaxed” and “loose” detection strictness levels are distributed in 2,129 different species (supplementary table S1, Supplementary Material online). To classify these pairs as either chromosomal, plasmidic, or viral, we retrieved the prophage regions (genomes of lysogenic phages inserted within host genomes) predicted within QSS-encoding chromosomes and plasmids present in the PHASTER database (Arndt et al. 2016). Then, if the genomic coordinates of a candidate QSS were found to fall within a prophage region, we classified this QSS as viral instead of bacterial. Of the 12,050 strict pairs, we found that 9,545 are chromosomal (on 2,965 distinct chromosomes), 964 are plasmidic (on 677 distinct plasmids), 28 are observed within sequenced phage genomes (on 18 distinct genomes), and 1,523 were assessed by PHASTER as belonging to 1,383 distinct prophages (638 assessed as intact, 292 as questionable, and 453 as incomplete and thus presumably domesticated by the bacterial host [Bobay et al. 2014]) (supplementary table S1, Supplementary Material online). The 48,800 more hypothetical pairs are distributed across 7,915 distinct chromosomes, 504 distinct plasmids, 136 distinct sequenced phage genomes, and 692 distinct prophages (supplementary table S1, Supplementary Material online).

This unprecedented massive library of bacterial, plasmidic, and viral candidate communication systems represents a great potential for expanding our knowledge of density-dependent processes within microbial communities.

### Identification of 21 Novel High-Confidence Subfamilies of Candidate RRNPP QSSs

**Fig. 3.**
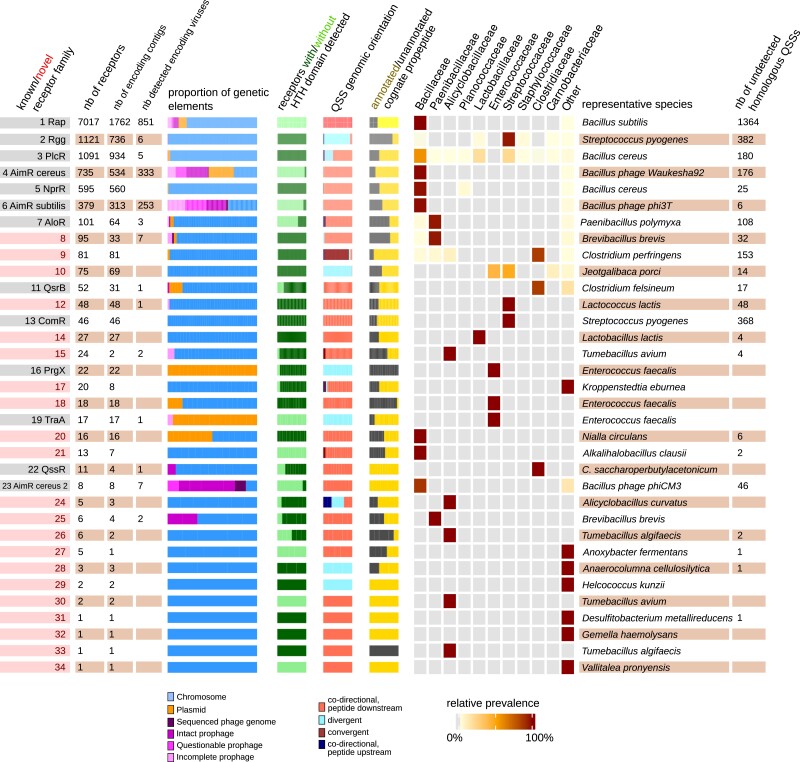
Size, functional characteristics, MGE distribution, and taxonomic representation of the 34 high-confidence clusters of candidate RRNPP QSSs. On this figure, rows represent genetic clusters of candidate RRNPP QSSs predicted at a “strict” detection strictness level and are ordered by cluster size. Each column corresponds to a cluster's characteristic. Column 1: known or novel cluster. Column 2: number of detected candidate receptors. Column 3: number of detected encoding elements. Column 4: number of detected encoding viral elements. Column 5: proportion of encoding chromosomes, plasmids, and temperate phages. Column 6: proportion of candidate receptors with a detected N-terminal HTH DNA-binding domain indicative of a transcription factor activity (dark green). Of note, some receptors like AimR can have an HTH domain but can still not be matched by public HMMs of HTH. Column 7: proportion of QSSs found in the different genomic orientation. Column 8: proportion of QSSs with and without an annotated candidate cognate propeptide. Column 9: Distribution and prevalence across taxonomic families. Column 10: representative species. Column 11: number of additional QSSs detected by the expansion_to_homologs search module of RRNPP_detector.

To facilitate the exploration of this library, we sought to classify into clusters the detected pairs (irrespective of their detection strictness level), based on the sequence similarity of the receptors. We designed our clustering method such that a cluster would correspond as closely as possible to the definition of a subfamily of RRNPP receptors, using the 11 already known subfamilies of receptors described in the literature as a baseline for testing (see Materials and Methods). In the method chosen for this task, the sequences of receptors are Blasted again each other, resulting in a weighted sequence similarity network in which the Markov Clustering Algorithm (MCL algorithm) identifies natural clusters by exploiting the property that random walks on a network will infrequently go from one natural cluster to another (Enright et al. 2002) (more details in Materials and Methods). Applying this algorithm to all hypothetical receptors, 307 natural clusters were identified, of which 76 contained at least one receptor forming a QSS detected at the “strict” level. We then filtered down these 76 clusters to 34 high-confidence clusters in which 1) the domain architecture of receptors complies with that of reference RRNPP receptors, 2) the amino-acid profiles of cognate propeptides comply that of reference RRNPP propeptides, and 3) the size of the cluster is consistent with the number of sequenced genomes for the taxonomic range of encoding taxa (supplementary table S2, Supplementary Material online; Materials and Methods). With this filtering procedure, the 12,050 “strict” pairs were narrowed down to 11,872 “strict” pairs (supplementary table S2, Supplementary Material online). An overview of the size, the functional characteristics, the MGE distribution, and the taxonomic distribution of the candidate RRNPP QSSs from these 34 high-confidence clusters is given in figure 3. As desired, each of the 11 already known Rgg, Rap, NprR, PlcR, PrgX, TraA, AimR, ComR, AloR, Qsr, and QssR receptor subfamilies was depicted by a single cluster, with the exception of the AimR subfamily, in which communication systems encoded by phages of the *B. cereus* group were grouped in two clusters whereas those encoded by phages of the *B. subtilis group* were grouped in one cluster. Hence, only 13 clusters of the 34 identified clusters were already known prior to this study. In other words, with the “strict” output only, our results may represent more than a 2-fold expansion of the described genetic diversity of RRNPP QSSs. The similarity distance matrix of these 34 clusters and the phylogeny of the closest, alignable clusters are displayed in supplementary figure S2, Supplementary Material online. Finally, we report 16 additional clusters composed of “relaxed” and/or “loose” pairs that also satisfy the three aforementioned filtering criteria and encompass propeptides which, although not asserted as carrying a SEC-secretion tag, had a Sec/SPI likelihood score >0.2 and aRBS motif of high confidence (bin > 13) (supplementary table S3, Supplementary Material online).

### A Treasure Trove of Peptide-Based Communication Codes

**Fig. 4.**
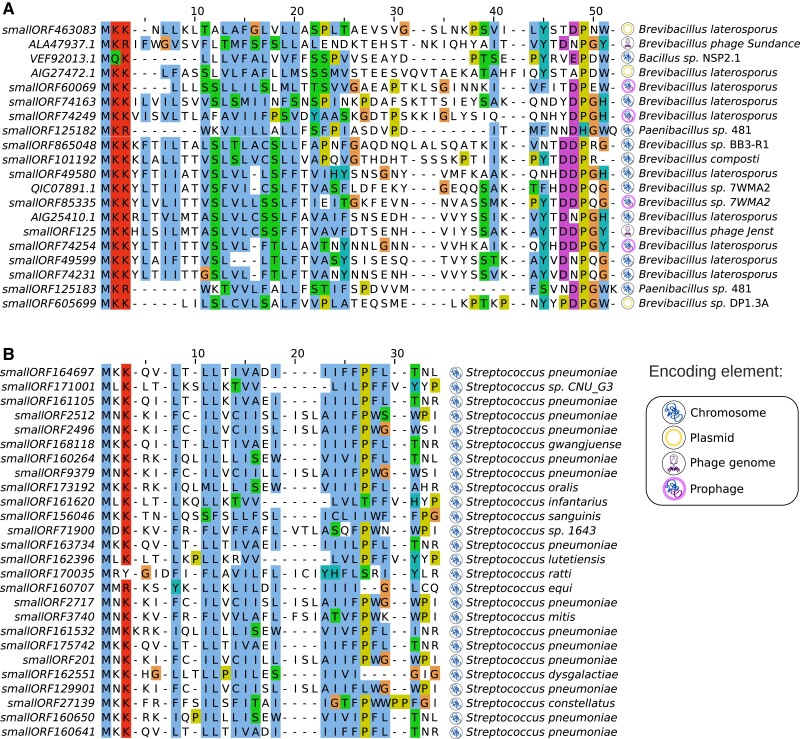
MSAs of candidate propeptides associated with novel clusters of receptors corresponding to different detection strictness levels. For each alignment, residues are colored according to the “Clustal” color code based on their physicochemical properties (see http://www.jalview.org/help/html/colourSchemes/clustal.html). The canonical amino-acid profile of RRNPP propeptides involves an N-terminal signal sequence for a secretion system composed of a short basic domain, followed by a longer hydrophobic region. The C-terminal region is composed of cleavage sites for membrane-bound peptidase and/or exopeptidase. Although there are exceptions to this trend (e.g., in the NrpX propeptides), the last four to ten residues at the C-terminal usually correspond to the mature communication peptide. (*A*) Sampled “strict” propeptides encoded by chromosomes, plasmids, or phage genomes associated with the cluster 8 (*Paenibacillaceae* family). (*B*) Sampled “relaxed” and “loose” chromosomal propeptides from *Streptococcus* bacteria associated with the known Rgg cluster.

To gain insights on the potential of these data to unravel novel communication codes, we analyzed the sequences of sampled candidate propeptides from all the 34 high-confidence clusters (supplementary fig. S1, Supplementary Material online). As expected, the “strict” candidate propeptides associated with these clusters harbor the canonical properties of RRNPP propeptides (specific example of novel cluster 8 identified in chromosomes, plasmids, and phages of *Paenibacillaceae* bacteria in fig. 4*A*, overview of the 34 clusters in supplementary fig. S1, Supplementary Material online), with a small N-terminal basic region, a central hydrophobic region, and a C-terminus that usually correspond to the mature communication peptide released by membrane-bound peptidases and/or exopeptidases (Pottathil and Lazazzera 2003; Erez et al. 2017; Bernard et al. 2020). On this basis, the array of propeptides, both within known and novel clusters, show great promise for the discovery of novel communication codes.

We then explored the potential of the “relaxed” and “loose” outputs of RRNPP_detector to predict RRNPP QSSs involving a noncanonical SEC-dependent secretion of their propeptides. To this end, we focused on the well-described Rgg subfamily (high-confidence cluster 2), because the cognate SHP propeptides of Rgg receptors are known to be exported by the PptAB translocon (Aggarwal et al. 2014) and are consistently not recognized by SignalP as being exported via the SEC translocon (fig. 1*B*). Accordingly, only propeptides adjacent to Rgg receptors matching the HMM of canonical SHP propeptides are placed in the “strict” output of RRNPP_detector. Yet, other PptAB-secreted propeptides that have an amino-acid profile different from that of reference SHPs might be genuine QS propeptides. With this respect, figure 4*B* shows that “relaxed” and “loose” propeptides found in the chromosomes of *Streptococcus* bacteria that are not matched by the HMM of SHPs but whose cognate receptors nonetheless belong to the Rgg cluster can harbor N-terminal basic residues followed by a hydrophobic region that likely corresponds to a signal sequence for the PptAB translocon. Hence, these candidate propeptides may be divergent variants of canonical SHP propeptides and illustrate that the “relaxed” and “loose” outputs of RRNPP_detector can be relevant to identify candidate functional QS propeptides undergoing alternative secretory processes.

According to these observations, our collection of “strict,” “relaxed,” and “loose” RRNPP QSSs may represent a real treasure trove of communication codes awaiting functional characterization. Thus, to foster these discoveries, we made the whole data set of candidate RRNPP propeptides publicly available at: https://github.com/TeamAIRE/RRNPP_candidate_propeptides_exploration_dataset/raw/main/RRNPP_candidate_propeptides_exploration_dataset.zip.

### Some Plasmids and Bacteriophages Encode Multiple Communication Systems Belonging to Distinct RRNPP Subfamilies

Interestingly, the distribution of “strict” candidate RRNPP QSSs from the 34 high-confidence clusters across chromosomes, plasmids, and phage genomes revealed that despite the important metabolic cost associated with RRNPP communication systems (Dogsa et al. 2021), many genetic elements can encode multiple QSSs (fig. 3). If the presence of multiple QSSs on a single chromosome is not rare (Do and Kumaraswami 2016; Even-Tov et al. 2016b; Voichek et al. 2020; Gallegos-Monterrosa et al. 2021) due to the selective pressure that may exist for the acquisition of a QSS with a novel peptide–receptor specificity (Even-Tov et al. 2016a, 2016b; Kalamara et al. 2018), only two phage genomes were previously reported to encode two RRNPP QSSs (Bernard et al. 2020). Here, we identified 263 “multilingual” MGEs, which represent 12.65% of the MGEs predicted to encode at least one QSS. Indeed, we found more than one “strict” candidate RRNPP QSSs in 181 plasmids (up to nine QSSs in the megaplasmid pYC1 of *Bacillus thuringiensis* YC-10), in six sequenced genomes of *Bacillus* phages (all encoding two QSSs), and in 76 *Bacillus* prophages (up to three in an intact prophage of *Bacillus licheniformis* CP6) (supplementary table S4, Supplementary Material online). If some MGEs encode multiple copies of a same cluster of RRNPP QSSs, like the aforementioned *B. licheniformis* prophage that carries three variants from the Rap–Phr cluster, some MGEs were found to encode QSSs belonging to distinct clusters. In particular, we identified the combination of the AimR–AimP system with the Rap–Phr system in 67 *Bacillus* (pro)phages, a combination that we previously described only in two (pro)phages (Bernard et al. 2020). The Rap and PlcR clusters were also found co-occurrent within ten plasmids. Interestingly, if almost all of the detected “multilingual” MGEs were associated with hosts from the *Bacillus* genus, we also found plasmids encoding two QSSs within *Priestia koreensis* FS-1 (two NprR–NprX systems) and *Brevibacillus laterosporus* LMG 15,441 (one AloR–AloP system and a novel candidate system corresponding to cluster 8 [fig. 4*A*]) (supplementary table S4, Supplementary Material online). Overall, the presence of multiple communication systems within MGEs might confer upon these entities a high adaptability to changes in their social context.

### Some Subfamilies of RRNPP Communication Systems Are Found Across Chromosomes, Plasmids, and Phages

**Fig. 5.**
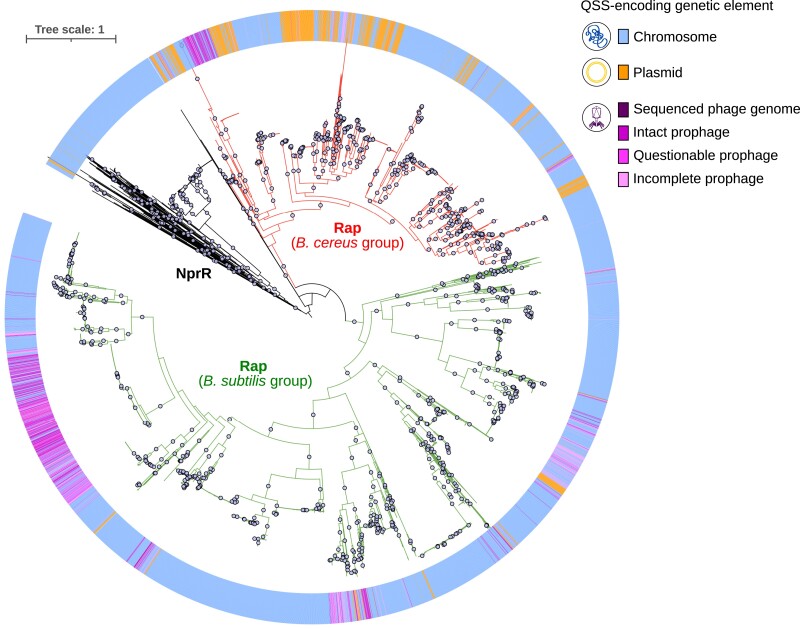
Polyphyly of viral Rap–Phr systems. The figure displays the maximum-likelihood phylogenetic tree of the detected receptors from the Rap cluster (no DNA-binding domain) and the detected receptors from the NprR cluster (DNA-binding domain). NprR was used as an outgroup for rooting the tree, consistently with the common phylogenetic of the Rap and NprR subfamilies (Perchat et al. 2016). Gray dots indicate branches supported by >90% bootstraps. Branch lengths are proportional to the expected number of substitution per site, as indicated by the scale bar at the top left. The color strip surrounding the phylogenetic tree assigns a color to each receptor forming a QSS based on the type of encoding genetic element: blue for chromosomes, orange for plasmids, and dark purple for sequenced genomes of temperate bacteriophages, different levels of purple for PHASTER-predicted intact, questionable, and incomplete prophages. QSSs encoded by incomplete prophages may be indicative of a capture of a viral QSS by a host, as a result of prophage domestication by the host genome (Bobay et al. 2014).

From a host–MGE coevolution perspective, it is also interesting to note that 14 clusters of candidate RRNPP QSSs are found to be shared between chromosomes and MGEs, which highlight that some communication systems may be externalized between chromosomes, plasmids, and phages (Corel et al. 2018) (fig. 3). If this feature has previously been reported for already known clusters of RRNPP QSSs (Bernard et al. 2020; Felipe-Ruiz et al. 2022), it is interesting to note that this can also be the case for certain novel candidate subfamilies (figs. 3 and 4*A*). Here, we took advantage of the large amount of viral and plasmidic QSSs within the Rap–Phr cluster to investigate in more details the evolutionary dynamics that may underlie the distribution of communication systems across distinct genetic elements. To this end, we inferred the phylogeny of Rap receptors forming a detected Rap–Phr system and looked at the distribution of the chromosomal, plasmidic, and viral members in this phylogeny. Remarkably, the Rap–Phr QSSs encoded by phages and plasmids appeared highly polyphyletic, hinting at multiple independent acquisitions of this communication system within MGEs. Overall, our observations suggest that bacteria, plasmids, and phages can frequently exchange communication systems (fig. 5).

### Clues That Phage and Prophage-Encoded QSSs May Regulate Bacterial Behaviors as a Function of (Pro)Phage Densities

The existence of homologous QSSs found in chromosomes and MGEs also implies that some QSSs may interfere with the regulation of target genes in different genomes/genetic elements, which may notably give rise to density-dependent manipulations of bacterial hosts by MGEs, as shown for non-RRNPP QSSs in Silpe and Bassler (2019). In this regard, the case of the previously analyzed Rap–Phr cluster is interesting because in *Bacillus* bacteria, Rap is a well-known protein inhibitor of ComA and/or Spo0F-P, which are key activators of the competence and sporulation pathways, themselves linked to processes as important as biofilm formation, cannibalism, and public good production (Schultz et al. 2009, 2013; González-Pastor 2011; Kalamara et al. 2018). When the encoding subpopulation is small, cheating or vegetative growth is advantageously promoted through Rap-mediated inhibition of ComA-P/SpoF-P, when larger subpopulations of *Bacillus* bacteria either produce public goods or commit to sporulation and leave nutrients available (González-Pastor 2011; Pollak et al. 2016; Kalamara et al. 2018). However, when the Rap–Phr encoding subpopulation gets larger and is no longer in minority in the local neighborhood, the Rap-mediated inhibition of cheating/sporulation becomes detrimental and is alleviated by the inhibition of Rap by its cognate Phr communication peptide (Pollak et al. 2016; Kalamara et al. 2018). Here, the detected presence of Rap–Phr systems on 1,531 MGEs suggest that this dynamic regulation of competence/sporulation may sometimes be dependent on a local density of MGEs rather than on a genuine density of bacterial cells, as shown for plasmids in Cardoso et al. (2020) and as hinted in prophages by the observed inhibition of Spo0F-P upon heterologous expression of a prophage-encoded Rap receptor (Even-Tov et al. 2016a; Bernard et al. 2020).

**Fig. 6.**
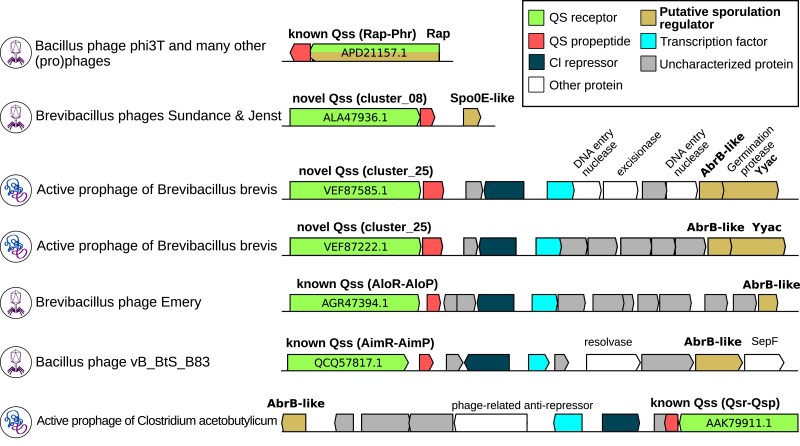
Multiple occurrences of a putative density-dependent sporulation-hijacking genomic signature in various temperate bacteriophages of *Firmicutes*. Each genomic context highlights a candidate “strict” RRNPP QSSs with a putative adjacent target regulon comprising a viral homolog of a bacterial sporulation initiation regulator (either Rap, Spo0E, or AbrB) in a sequenced phage genome (virion icon) or in a prophage (lysogenized chromosome icon). Genes are colored according to their functional roles, as displayed in the legend. For each candidate QSS, the corresponding “strict” RRNPP cluster as well as the NCBI id of the receptor are displayed. The five last genomic contexts correspond to an “RRNPP QSS—divergently transcribed c1 repressor—potential operon of codirectional genes” configuration, which has been shown to be indicative of a regulation of the operon by the viral RRNPP QSS in Stokar-Avihail et al. (2019).

Surprisingly, the analysis of viral candidate QSSs from the “strict” data set suggested that Rap–Phr systems may not be the only viral QSSs associated with bacterial sporulation modulation. Indeed, we identified additional viral RRNPP QSSs belonging to distinct clusters with a putative sporulation-hijacking genomic signature. This prediction lies on the observation that their receptor harbors a DNA-binding domain and thus likely regulates the expression of adjacent genes (a trend especially true in MGEs [Erez et al. 2017; Neiditch et al. 2017; Kohler et al. 2019; Stokar-Avihail et al. 2019], as shown in fig. 1) and that a viral homolog of the bacterial *spo0E* or *arbB* sporulation regulator is found adjacent of the QSSs (fig. 6). The same genomic context, albeit not encoded by a phage, was shown to underlie a density-dependent regulation of the *spo0E*-like gene by the adjacent QSS in *Paenibacillus polymyxa* (Voichek et al. 2020). As a matter of fact, the Spo0E, AbrB, and Rap proteins form a decision-making circuit that controls the timing of sporulation in *Bacilli* by regulating the accumulation of Spo0A-P, the master activator of the sporulation initiation pathway (Shafikhani and Leighton 2004; Fujita and Losick 2005; Schultz et al. 2009, 2013). ⁠On this basis, if the aforementioned (pro)phage-encoded *spo0E*-like or *abrB*-like genes were genuine sporulation regulators, the (pro)phage-(pro)phage communication systems predicted to control the expression of these genes could influence SpoA-P accumulation dynamics within the hosts and thereby dynamically manipulate the sporulation initiation pathway. With regard to this hypothesis, it is interesting to mention that a mutant for the putative receptor encoded by the intact prophage of *C. acetobutylicum* ATCC 824 presented in figure 6 was experimentally shown to produce three times less endospores than the wild-type after 7 days of culture (Kotte et al. 2020).

### Identification of 196 BGCs Inferred to Be Regulated by an Adjacent Candidate RRNPP QSS

**Fig. 7.**
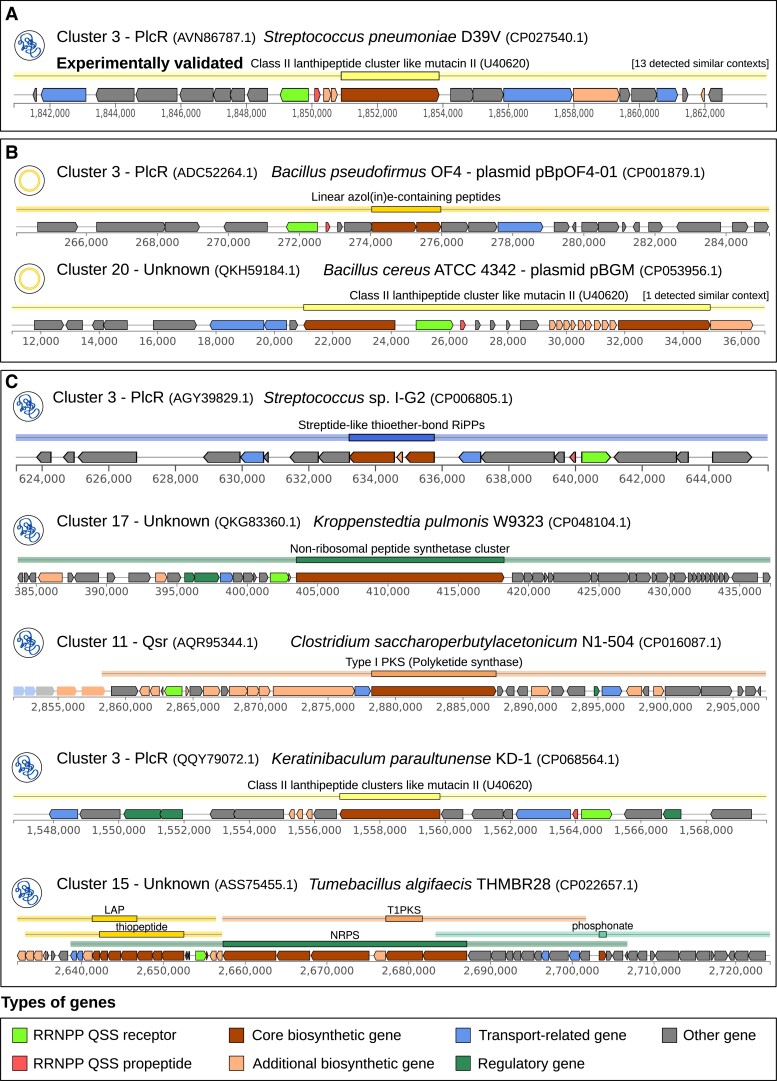
Selection of BGCs inferred to be regulated by an adjacent “strict” candidate RRNPP QSS. For each genomic context, a thumbnail indicates the genetic element encoding the BGC region (chromosome or plasmid) and is followed by the name of the cluster of the candidate RRNPP QSS and the NCBI ID of its receptor. Finally, the name of the encoding genome along with its NCBI accession is given. The second line indicates the biosynthesis mode of the BGC, as classified by antiSMASH. The tick marks at the bottom of each BGC correspond to genomic coordinates (in bp). (*A*) Proof of concept provided by a BGC demonstrated to be regulated by a RRNPP QSS in Hoover et al. (2015) and captured by our method. (*B*) Plasmidic BGCs inferred to be regulated by a candidate RRNPP QSS. (*C*) Small overview of chromosomal BGCs inferred to be regulated by a candidate RRNPP QSS.

In addition to these fundamental aspects of bacteria–MGE coevolution, RRNPP QSSs may also regulate adaptive bacterial traits of applied interest, such as the production of public good metabolites, for example antimicrobial compounds, because only a collective production may bring such molecules to the concentration levels required to exert a significant effect on the microbial community (Heilmann et al. 2015; Palmer and Foster 2022). Consistent with the fact that QSSs in which the receptor is a one component tends to regulate adjacent genes (fig. 1; Engebrecht et al. 1983; Brotherton et al. 2018; He et al. 2018), many biosynthetic gene clusters (BGCs) that produce antimicrobials have been demonstrated to be controlled by a QSS located in their genomic vicinity, be it a small molecule-based (Brotherton et al. 2018; He et al. 2018) or a peptide-based QSS (fig. 7*A*; Hoover et al. 2015; Rued et al. 2021). As a major challenge in the field of natural product discovery is that many BGCs are not expressed under laboratory growth conditions (Rutledge and Challis 2015), identifying BGCs regulated by an adjacent QSS may be promising as their link with population density provides some understanding about how to elicit their production in the laboratory. Hence, to identify candidate QSS-regulated BGCs, we first searched for BGCs with antiSMASH standalone version 6.0.0 (default parameters) (Blin et al. 2021) in the genetic elements encoding a “strict” candidate RRNPP QSS with a receptor detected as a transcription factor (harboring an HTH DNA-binding domain). We then intersected the list of the BCGs detected by antiSMASH with our list of candidate RRNPP QSSs on the basis of the inclusion of the QSS region (from the start codon of the first gene to the stop codon of the second gene) within the region of a BGC defined by antiSMASH. This resulted in a subset of 196 candidate BGCs potentially under control of an RRNPP QSSs, distributed in the *Alicyclobacillaceae* (*n* = 10), *Bacillaceae* (*n* = 25), *Paenibacillaceae* (*n* = 10), *Staphylococcaceae* (*n* = 5), *Thermoactinomycetaceae* (*n* = 4), *Carnobacteriaceae* (*n* = 2), *Lactobacillaceae* (*n* = 6), *Streptococcaceae* (*n* = 125), *Clostridiaceae* (*n* = 7), *Peptoniphilaceae* (*n* = 1), and *Tissierellaceae* (*n* = 1) taxonomic families (supplementary table S5, Supplementary Material online, and fig. **7**). Among these putative QSS-regulated BGCs, six are plasmidic, of which five are inferred by antiSMASH to produce antimicrobial peptides (fig. 7*B*). As these plasmidic RRNPP QSSs likely enact the production of defense metabolites only when the quorum of plasmids is met, these QSSs might create a selective pressure for the acquisition of the plasmid by host cells at high plasmid densities, supporting complex scenarios of coevolution. Interestingly, the 196 putative QSS-regulated BGCs produce major classes of natural products (supplementary table s5, Supplementary Material online, and fig. 7*C*), including ribosomally synthesized and posttranslationally modified peptides (RiPPs), nonribosomal peptides, and polyketides. Worth to note, RiPPs are of most frequent occurrence, likely reflecting the important roles of RiPPs in bacterial physiology (Li and Rebuffat 2020).

## Discussion

We predicted a wide range of novel candidate RRNPP QSSs in chromosomes or in MGEs (e.g., plasmids and phages) of *Firmicutes* using a computational approach that does not rely on sequence similarity search using known QSS proteins as queries (supplementary tables S1–S3, Supplementary Material online and figs. 2 and 3). This massive, publicly available library of candidate communication systems shows great promise for the characterization of many density-dependent mechanisms in bacteria, plasmids, and phages, with major fundamental and applied outcomes.

In this regard, we gave a few examples of how the prediction of QSSs can be coupled with functional insights, by exploiting the trend that the target regulon of an RRNPP QSS often lies in its genomic vicinity, a trend especially true for MGEs (Erez et al. 2017; Neiditch et al. 2017; Kohler et al. 2019; Stokar-Avihail et al. 2019). This allowed to highlight a putative convergent evolution of the functional association between QS-mediated phage–phage communication and bacterial sporulation manipulation, with nonhomologous viral QSSs from different bacteriophage species found adjacent to a viral homolog of one of the bacterial Rap, AbrB, or Spo0E sporulation regulator (fig. 6). If this association was experimentally validated, the fact that phages and/or prophages could communicate to decipher when it is the most evolutionary advantageous to manipulate host pathways would capture a novel feature of bacteria–phages coevolution, since the experimentally validated phage-encoded QSSs were thus far shown to only regulate viral processes such as the lysis–lysogeny decision (Erez et al. 2017). In addition, this validation could invite to reconsider the sporulation decision-making process as a biological process that may sometimes fall under the scope of a (pro)phage–host collective, rather a strict bacterial process of last resort, with important implications considering that the endospore is the transmissive form of many bacteria, be they commensal or pathogens for humans (Mallozzi et al. 2010; Postollec et al. 2012; Swick et al. 2016).

It is also interesting to mention that we found one of these putative host-hijacking QSS, the Rap–Phr system, in co-occurrence with the arbitrium system within 67 *Bacillus* phage genomes (supplementary table S4; Supplementary Material online). Although the arbitrium system regulates the lysis–lysogeny transition upon *Bacillus* infection, we previously hypothesized that prophage-encoded Rap–Phr systems might confer upon lysogenized hosts selective advantages over nonlysogenized hosts such as the evasion to public good production at low population densities, for the evolutionary benefit of the prophage–host collective (Bernard et al. 2020). In general, owning multiple QSSs regulating distinct biological processes might enable behavioral transitions according to different regimes of densities, reflected by the different quorums associated with each QSS (Mehta et al. 2009). In total, of the 2,078 MGEs within which at least one “strict” candidate QSS has been predicted, 263 were found to encode more than one QSS (supplementary table S4; Supplementary Material online). The observation that 12.65% of the QSS-encoding detected MGEs encode multiple QSSs generalizes the notion that phages and plasmids may subtly assess changes in their social context and adapt their evolutionary strategy accordingly. In light of the consideration that different QSSs owned by an MGE can be more or less conserved across nonkin MGEs, neighbors, or hosts, such “multilingual” MGEs could theoretically react to the density of multiple heterogeneous subpopulations to which these MGEs nonetheless always belong. Accordingly, encoding several QSSs more or less specific to its kins might enable an MGE to contextualize its own population density with respect to that of other heterogeneous populations.

In addition to these fundamental aspects of bacteria–MGE coevolution, a more applied example of the functional investigations conducted in this study was given by the identification of 196 BGCs of specialized metabolism inferred to be regulated by a candidate RRNPP QSS. Importantly, the predicted density-dependent expression of these BGCs hints at important adaptive ecological roles for the metabolites they produce. Thus, functional characterization of these BGCs may not only lead to the discovery of novel molecules of applied interest, such as novel antimicrobial molecules or candidate virulence factors to fight against, but could also be rich in lessons to better understand the lifestyle of their encoding species.

Overall, our analyses demonstrate that our methodology can unlock new biological knowledge regarding peptide-based biocommunication and can reveal novel density-dependent decision-making processes in bacteria, plasmids, and bacteriophages, with potential to enhance our understanding of microbial adaptation and bacteria–MGE coevolution. Yet, the communication systems described in this study likely do not represent the entire landscape of RRNPP QSSs. Indeed, we analyzed only complete genomes of *Firmicutes*, and many candidate RRNPP QSSs likely await to be unearthed in bacterial scaffolds, contigs, and metagenomics-assembled genomes. In this respect, it is important to mention that the *Firmicutes* phylum represents with Bacteroidetes the most prevalent phylum in human gut microbiomes (Manor et al. 2020). Accordingly, the application of our publicly available RRNPP_detector software against human-associated metagenomics-assembled genomes or MGEs (e.g., from the human MGE database [Lai et al. 2021] or the Gut Phage Database [Camarillo-Guerrero et al. 2021]) would be of high relevance to infer density-dependent behaviors that may take place within human intestinal microbiomes, plasmidomes, and viromes.

## Materials and Methods

### Definition of the RRNPP Signature

We carefully mined the literature to identify all experimentally validated RRNPP subfamilies and identify one functionally validated representative QSS for each subfamily (Pottathil and Lazazzera 2003; Aggarwal et al. 2014; Hoover et al. 2015; Do and Kumaraswami 2016; Shanker et al. 2016; Even-Tov et al. 2016a; Erez et al. 2017; Neiditch et al. 2017; Stokar-Avihail et al. 2019; Feng et al. 2020; Kotte et al. 2020; Voichek et al. 2020). We then fetched the sequences of the reference QSSs from the NCBI or the IMG database (NCBI Resource Coordinators 2016; Chen et al. 2021), visualized their genomic context, and analyzed their similarities to delineate decision rules for the detection by RRNPP_detector of candidate RRNPP QSSs at a “strict” detection strictness level. The results of these preliminary analyses are fully summarized in figure 1*B*. The extreme values in the lengths of the validated receptors (285–473aa) and propeptides (21–50aa) (fig. 1*B*) were used as references to define default ranges of lengths for candidate receptors (250–500aa) and propeptides (10–100aa). We chose an upper limit of 100aa for candidate propeptides due to the intragenic duplications reported to frequently happen in their coding sequences (Even-Tov et al. 2016a). Likewise, the extreme values of intergenic distances (−50 to 191 bp) between reference receptors and propeptides (fig. 1*B*) served as a baseline to define the default intergenic distance (−60 to 400 bp) to define a candidate receptor–propeptide pair. Using InterProScan (Jones et al. 2014), motif search reliant on the Pfam, Smart, Tigrfam, Superfamily, Panther, and Cath-Gene3D databases of HMMs was conducted against the protein sequences of the reference receptors to illustrate the fact that the publicly available curated libraries and superfamilies of HMMs of TPRs and DNA-binding domains matching these proteins are relevant to identify RRNPP receptors (fig. 1*B*). SignalP version *5.0b Linux x86_64* was run with the option “-org gram+” against the reference propeptides to illustrate the reliability of this software to predict the SEC-dependent secretion of quorum-sensing propeptides (Almagro Armenteros et al. 2019). Indeed, only the PrgQ and SHP reference propeptides were not predicted by SignalP to harbor a SEC-secretion tag (fig. 1), consistent with the fact that they are the only RRNPP propeptides mentioned to be exported via the alternative PptAB translocon (Neiditch et al. 2017).

### Constitution of the Library of HMMs of TPRs Used to Detect Putative RRNPP Receptors

The single HMMs (from the Smart, Pfam, and Tigrfam databases of HMMs) and superfamilies/collections of HMMs (from the Cath-Gene3d and Superfamily databases of HMMs) matching the sequences of RRNPP receptors (shown in fig. 1) were retrieved and compiled in a library of HMMs of TPRs. In addition, based on the observation that these generic HMMs were not sufficient to identify all AimR receptors described in Stokar-Avihail et al. (2019), we built an HMM from the entire multiple sequence alignment (MSA) of AimR receptors described in Erez et al. (2017), as well as an HMM corresponding to the conserved C-terminal TPRs in the MSA of AimR described in Stokar-Avihail et al. (2019) (MSAs are available in https://github.com/TeamAIRE/RRNPP_detector/tree/main/data/fasta). Finally, we included within this library the following additional HMMs of TPRs from the Pfam database: TPR_1 (PF00515.30), TPR_2 (PF07719.19), TPR_3 (PF07720.14), TPR_4 (PF07721.16), TPR_5 (PF12688.9), TPR_6 (PF13174.8), TPR_7 (PF13176.8), TPR_9 (PF13371.8), TPR_10 (PF13374.8), TPR_11 (PF13414.8), TPR_14 (PF13428.8), TPR_15 (PF13429.8), TPR_16 (PF13432.8), TPR_17 (PF13431.8), TPR_18 (PF13512.8), TPR_19 (PF14559.8), TPR_20 (PF14561.8), TPR_21 (PF09976.11), TPR_22 (PF18833.3), ComR_TPR (PF18710), and TPR_MalT (PF17874.3).

### RRNPP_Detector Algorithm

The minimal input of RRNPP_detector is a FASTA file of the nucleotide sequences of target genome(s), metagenomics-assembled genome(s) or contig(s). In this case, RRNPP_detector calls Prodigal to find coding sequences within these genomes and output their proteome(s) (Hyatt et al. 2010). It is also possible to directly submit a FASTA file of annotated protein sequences and a general feature format (GFF) file referencing the coordinates of their coding sequences in target genome(s). However, in this case, RRNPP_detector will be constrained to work only with annotated proteins. The best option is to provide the FASTA files corresponding to the target genome(s) and their corresponding annotated proteome(s), along with the annotations. At first, RRNPP_detector first reduces the search space by retaining only annotated proteins (provided by the user or detected by Prodigal) that have a length compatible with RRNPP receptors (by default 250–500aa) and propeptides (by default 10–100aa). Then, the aforementioned HMMs of TPRs are used as queries in an HMMSearch against the 250–500aa-long proteins to identify putative receptors (*E*-value < 1E−5, HMM coverage > 65%) (Eddy 2011). Optionally, if the user calls RRNPP_detector with the “—tprpred” option, the complementary TprPred software will be launched against the 250–500 proteins not detected by HMMsearch to identify additional TPR-containing putative receptors (Karpenahalli et al. 2007). These potential receptors next undergo a computational characterization step. First, a Blastp search (Altschul et al. 1990) of reference RRNPP receptors against these potential receptors is launched to identify the subset of candidate receptors with sequence similarity to known QSS receptors (by default: *E*-value ≤ 1E−5, identity ≥ 20%, and alignment coverage ≥ 60% of the length of both the query and the target sequences). In addition, an HMMSearch of DNA-binding domains found in reference receptors (PFAM: PF01381; Gene3D: 1.10.260.40; Superfamily: 47,413; SMART: SM00530) is launched to identify candidate receptors with a predicted transcription factor activity. Then, only annotated proteins of 10–100 found directly adjacent to these putative TPR-containing receptors are retained. In addition, if the nucleotide FASTA file of target genome(s) is available, RRNPP_detector will search for putative unannotated propeptides encoded in the flanking regions of each coding sequence of a candidate receptor, using a module we named Small Peptide with RBS Annotation Tool (SPRAT). SPRAT defines upstream and downstream flanking regions based on the minimal and maximal allowed intergenic distances between the receptor and the propeptide genes (by default −60 to 400 bp) and on the maximal length of the propeptide (by default 100aa, therefore 303 bp). Orfipy is then called against the flanking regions of each candidate receptor to identify any putative protein-coding sequence within the length boundaries of a candidate propeptide (by default, starting with the ATG start codon) (Singh and Wurtele 2021). Nested open reading frames (ORFs) are then identified by SPRAT (e.g., if an in-frame start codon is found within a detected ORF). To limit the risk of false positives associated with the detection of small genes, SPRAT takes advantage of the observation that the absence of a translation-initiation SD RBS motif upstream from a gene in *Firmicutes* can be considered as a strong predictor that the gene is likely not translated/functional, as more than 90% of the annotated protein-coding genes encoded by *Firmicutes* are preceded by a SD RBS (Omotajo et al. 2015). Accordingly, SPRAT leverages the 27 hierarchical regular expressions introduced by Prodigal to detect SD RBS motifs (referred as bins) upstream from the putative protein-coding small ORFs (from −21 to −1 bp to start codon). By default, SPRAT then exploits the results of the systematic analysis of RBS bin usage across prokaryotes led by Omatajo et al. to define the list of RBS motifs most predictive of a translation initiation (bins 27, 24, 23, 22, 20, 19, 16, 15, and 14). Accordingly, by default, only unannotated small peptides encoded in the vicinity of receptors that are preceded by these bins are retained. Subsequently, SignalP (and optionally PrediSi) is called to predict putative secretion tag within the remaining annotated and unannotated 10–100aa proteins (Hiller et al. 2004; Almagro Armenteros et al. 2019). If a protein is not predicted to harbor a SEC/SPI-secretion tag by SignalP and is adjacent to a Rgg receptor (corresponding to the TIGR01716 HMM of TPRs), an HMMSearch of the HMM of SHP propeptides is launched against it. All receptor–propeptide pairs with the propeptide being annotated or unannotated with an RBS bin > 13 that either matches the HMM profile of SHPs or is predicted to undergo a SEC/SPI-dependent secretion according to SignalP are then placed in the output folder associated with the “strict” detection strictness level. Optionally, RRNPP_detector can use the strict candidate receptors and propeptides as Blastp queries to detect homologous QSSs that did not pass the initial conservative thresholds. All remaining receptor–propeptide pairs in which the propeptide harbors any of the SP(Sec/SPI), TAT(Tat/SPI), or LIPO(Sec/SPII) signal sequence according to PrediSi or SignalP are placed in the output folder associated with the “relaxed” detection strictness level. Finally, the “loose” level outputs remaining TPR-containing putative receptors only if found adjacent to a peptide without a detected secretion tag but with an upstream SD RBS of high-usage across prokaryotes (bins 27, 24, 23, 22, 20, 19, 16, 15, 14, 13, 12, and 6), with the cognate peptide being chosen as the one with the highest RBS bin in the close genomic vicinity of the candidate receptor.

### Benchmarking

The known QSSs in the nine reference genomes were described in the studies referenced in table 1. The nine genomes were fetched from the NCBI and were given as input to RRNPP_detector, in a run with and without the default RBS filter to assess its impact on recall and precision. Reported hits in table 1 correspond to QSSs predicted at the “strict” detection strictness level and additional homologs identified by the “expansion_to_homologs” module of RRNPP_detector.

### Prophage Detection

When the NCBI genomic accession of a QSS-encoding element was present in the PHASTER database of already computed genomes, the corresponding prophage regions were retrieved. Each QSS was defined as viral if its genomic coordinates on a given chromosome/plasmid fell within a region predicted by PHASTER to belong to a prophage (qualified as either “intact,” “questionable,” or “incomplete” prophage).

### Clustering of QSSs

As a baseline for testing different clustering methods, we used the data set of AimR receptors from Stokar-Avihail et al. (2019) and a data set composed of receptors detected by RRNPP_detector in the *Bacillus* genomes from (Even-Tov et al. 2016a) and assessed by Blastp as close homologs of either Rap, PlcR, NprR, and AimR. Our goal was to define a method that would group each members of a subfamily of receptors into a single cluster, to convey the idea that one cluster = one genetic subfamily of QSSs. At first, we tried the three following clustering methods that have in common to start with an all-versus-all alignment, subsequently filtered by fixed cutoff(s): 1) nontransitive Mmseq2 clustering algorithm with sequence identity and alignment coverage cutoffs (Steinegger and Söding 2017), 2) connected components with sequence identity and alignment coverage cutoffs (Méheust et al. 2018), and 3) EFI-EST–connected components with a Blastp *E*-value cutoff (Zallot et al. 2019). By varying the different cutoffs for each method, we observed that no sets of parameters successfully resulted in clusters matching the definition of RRNPP subfamilies in the literature. In contrast, we found that the MCL algorithm (Enright et al. 2002) that does not rely on fixed cutoffs for clustering but rather identify natural clusters based on simulation of stochastic flow in weighted graphs successfully assigned a single cluster to each of the AimR, Rap, PlcR, and NprR subfamilies. In a nutshell, the MCL algorithm, which is notably integrated in the pipeline of famous orthology inference methods (Li et al. 2003; Emms and Kelly 2019), finds cluster structure by exploiting the propriety that random walks on a graph will infrequently go from one natural cluster to another, based on graph transition probability estimates. MCL was applied as follows: 1) Blastp all versus all of the receptors, 2) application of a -log10 transform to the *E*-values of each pairwise alignment, with a ceiling set to 200 for any *E*-value below 1e−200; 3) weight normalization by the minimal percentage of the coverage between two proteins; 4) application of MCL to the resulting weighted sequence similarity graph with an inflating parameter of 1.4. Typically, the inflation affects the granularity or resolution of the clustering outcome, with low values (1.3, 1.4) leading to fewer and larger clusters and high values (5, 6) leading to more and smaller clusters. For protein family detection, an inflating parameter of 1.3 or 1.4 is recommended.

### Cluster Filtering

For each cluster, a search of randomly chosen receptors against the conserved domain database of the NCBI was used to discard clusters based on the detection of suspicious domain architecture, for example, proteins harboring suspicious domains not found in reference RRNPP receptors or N-terminal position of the TPR region as opposed to C-terminal, etc.…. Then, clusters for which the associated propeptides have an amino-acid profile different from that of reference RRNPP propeptides (basic N-terminal residues, long hydrophobic central region) were discarded. Finally, we accounted for sampling bias to retain small clusters only if encoded by species with a low number of sequenced genomes available (e.g., *Gemella haemolysans*).

### Identification of Already Known Clusters of Homologous Receptors

A Blastp⁠ search was launched using as queries the RapA (NP_389125.1), NprR (WP_001187960.1), PlcR (WP_000542912.1), Rgg2 (WP_002990747.1), AimR (APD21232.1), AimR-like (AID50226.1), PrgX (WP_002366018.1), TraA (BAA11197.1), AloR13 (IMG: 2547357582), QsrB (AAK78305.1), QssR5 (AGF59421.1), and ComR (ADX23594.1) reference receptors and as a target database, the receptors from the high-confidence clusters. If the best hit of a reference RRNPP-type receptor gave rise to a sequence identity ≥ 30% over at least 80% mutual coverage, then the cluster to which this best hit belonged was considered as an already known cluster.

### Phylogenetic Tree of NprR and Rap Receptors

An MSA of the protein sequences of the NprR and Rap receptors forming a complete QSS was performed using MAFFT *version v7.453* (Katoh et al. 2002). The MSA was then given as input to IQ-TREE *version multicore 1.6.10* to infer a maximum-likelihood phylogenetic tree under the LG + G model with 1,000 ultrafast bootstraps (Nguyen et al. 2015). The tree was further edited via the Interactive Tree Of Life (iTOL) online tool (Letunic and Bork 2019).

### Identification of Putative BGCs Regulated by an Adjacent Candidate RRNPP QSS

BGCs were searched with antiSMASH standalone version 6.0.0 (default parameters) (Blin et al. 2021) in the 937 genetic elements encoding at least one candidate RRNPP QSS with a receptor detected as a transcription factor (matched by an HMM of an HTH DNA-binding domain). We then intersected the list of the 5,893 BCGs detected by antiSMASH with our list of candidate RRNPP QSSs encoded by these 937 genetic elements on the basis of the inclusion of the QSS region (from the start codon of the first gene to the stop codon of the second gene) within the region of a BGC defined by antiSMASH (region that extends slightly beyond the BGC itself).

## Supplementary Material

msad062_Supplementary_Data

## Data Availability

RRNPP_detector is freely available on GitHub online (https://github.com/TeamAIRE/RRNPP_detector). The database of candidate QSSs described in this study along with the sequences of the candidate propeptides is publicly available on GitHub online (https://github.com/TeamAIRE/RRNPP_candidate_propeptides_exploration_dataset/raw/main/RRNPP_candidate_propeptides_exploration_dataset.zip).
