# Supplementary material for: Large-Scale Identification of Known and Novel RRNPP Quorum-Sensing Systems by RRNPP_Detector Captures Novel Features of Bacterial, Plasmidic, and Viral Coevolution
Source: Mol Biol Evol. 2023 Mar 17;40(4):msad062. doi: 10.1093/molbev/msad062 (PMC10075063; doi:10.1093/molbev/msad062)
Supplement: msad062_Supplementary_Data [file msad062_supplementary_data.zip › supplementary_figures.pdf]

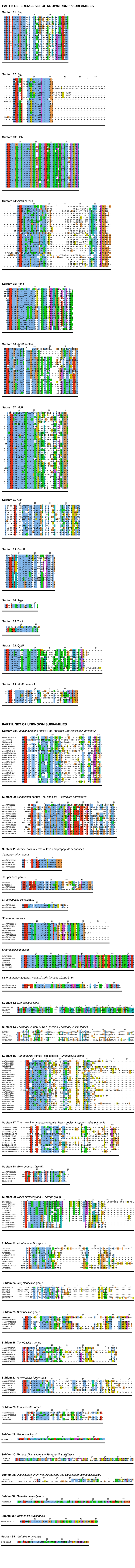
PART I: REFERENCE SET OF KNOWM RRNPP SUBFAMILIES
Subfam 01: Rap
Subfam 02: Rgg
Subfam 03: PlcR
Subfam 04: AimR cereus
Subfam 05: NprR
Subfam 06: AimR subtilis
Subfam 07: AloR
Subfam 11: Qsr
Subfam 13: ComR
Subfam 16: PrgX
Subfam 19: TraA
Subfam 22: QssR
Subfam 23: AimR cereus 2
PART II: SET OF UNKNOWM SUBFAMILIES
Subfam 08: Paenibacillaceae family; Rep. species: Brevibacillus laterosporus
Subfam 09: Clostridium genus; Rep. species: Clostridium perfringens
Subfam 11: diverse both in terms of taxa and propeptide sequences
Carnobacterium genus
Jeotgalibaca genus
Streptococcus constellatus
Streptococcus suis
Enterococcus faecium
Listeria monocytogenes Rev2, Listeria innocua 2015L-6714
Subfam 12: Lactococcus lactis
Subfam 14: Lactococcus genus; Rep. species: Lactococcus intestinalis
Subfam 15: Tumebacillus genus; Rep. species: Tumebacillus avium
Subfam 17: Thermoactinomycetaceae family; Rep. species: Kroppenstedtia pulmonis
Subfam 18: Enterococcus faecalis
Subfam 20: Nialla circulans and B. cereus group
Subfam 21: Alkalihalobacillus genus
Subfam 24: Alicyclobacillus genus
Subfam 25: Brevibacillus genus
Subfam 26: Tumebacillus genus
Subfam 27: Anoxybacter fermentans
Subfam 28: Eubacteriales order
Subfam 29: Helcoccus kunzii
Subfam 30: Tumebacillus avium and Tumebacillus algifaecis
Subfam 31: Desulfitobacterium metallireducens and Desulfosporosinus acidiphilus
Subfam 32: Gemella haemolysans
Subfam 33: Tumebacillus algifaecis
Subfam 34: Vallitalea pronyensis

Figure S1. Multiple Sequence Alignments of sampled 'strict' candidate pro-peptides from the 34 high-confidence clusters

For each alignment, residues are colored according to the "Clustal" colourcode based on their physico-chemical properties (see: http://www.jalview.org/help/html/colourSchemes/clustal.html). The canonical amino-acid profile of RRNPP pro-peptides involves a N-terminal signal sequence for a secretion system composed of short basic domain, followed by a longer hydrophobic region. The C-terminal region is composed of cleavage sites for membrane-bound and/or exo-peptidase. Although there are exceptions to this trend (e.g. in the NrpX propeptides), the last 4-10 last residues at the C-terminal usually correspond to the mature communication peptide. Of note it has been reported that a pro-peptide can harbor several and different mature communication peptide regions as a result of intragenic duplications, a means to quickly acquire a novel communication peptide for private communication. The figure is divided into two sets: i) MSAs corresponding to known clusters and ii) MSAs corresponding to novel clusters. We encourage to visit the full dataset at: https://github.com/TeamAIRE/RRNPP_candidate_propeptides_exploration_dataset (metadata and propeptide sequences available)

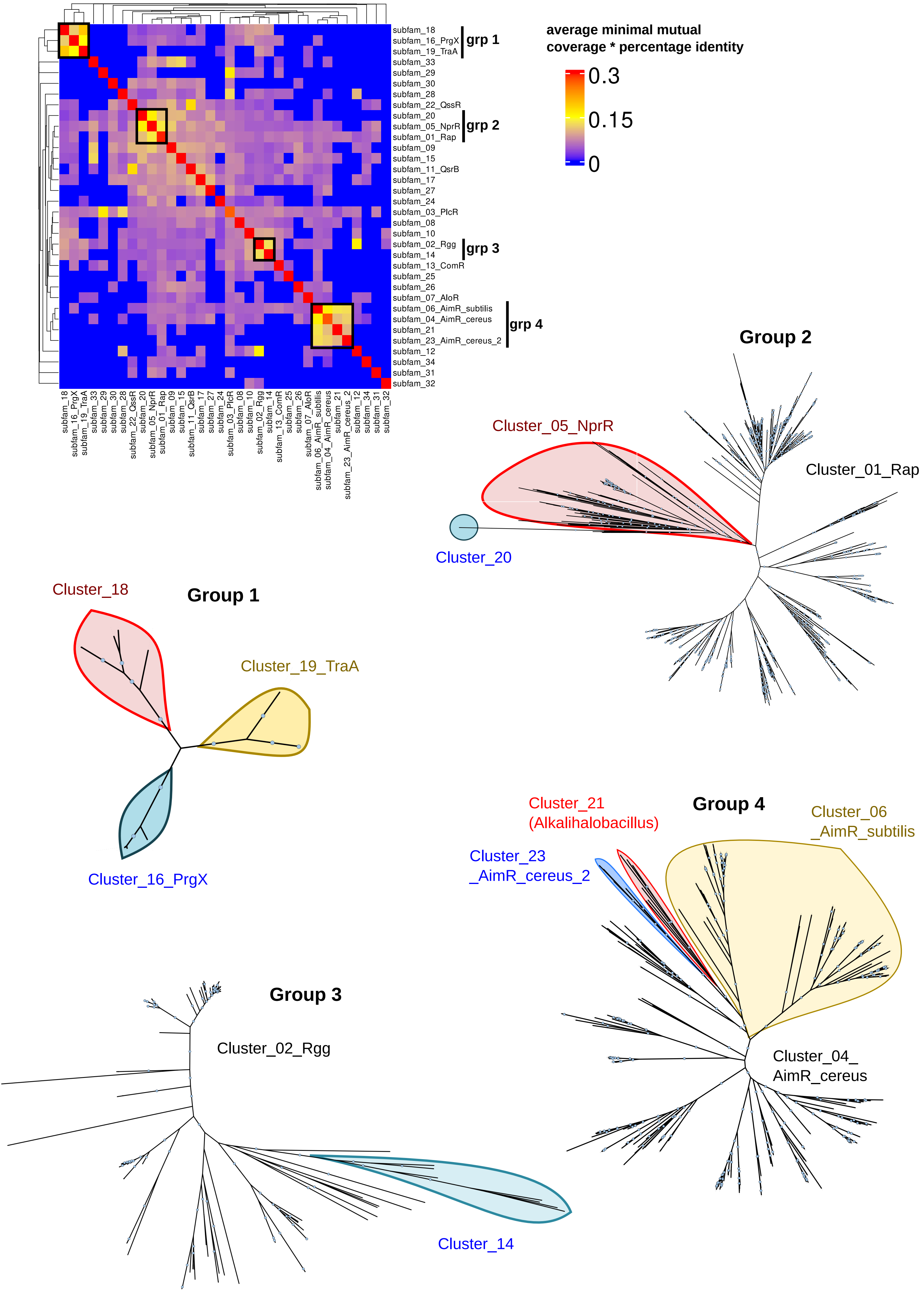
subfam_18
subfam_16_PrgX
subfam_19_TraA
subfam_33
subfam_29
subfam_30
subfam_28
subfam_22_QssR
subfam_20
subfam_05_NprR
subfam_01_Rap
subfam_09
subfam_15
subfam_11_QsrB
subfam_17
subfam_27
subfam_24
subfam_03_PlcR
subfam_08
subfam_10
subfam_02_Rgg
subfam_14
subfam_13_ComR
subfam_25
subfam_26
subfam_07_AloR
subfam_06_AimR_subtilis
subfam_04_AimR_cereus
subfam_21
subfam_23_AimR_cereus_2
subfam_12
subfam_34
subfam_31
subfam_32
grp 1
grp 2
grp 3
grp 4
average minimal mutual
coverage * percentage identity
0.3
0.15
0
Group 1
Cluster_18
Cluster_19_TraA
Cluster_16_PrgX
Group 2
Cluster_05_NprR
Cluster_01_Rap
Cluster_20
Group 3
Cluster_02_Rgg
Cluster_14
Group 4
Cluster_21
(Alkalihalobacillus)
Cluster_06
_AimR_subtilis
Cluster_23
_AimR_cereus_2
Cluster_04_
AimR_cereus

Figure S2. A) Distance between clusters. From the all vs all alignments of the 'strict' receptors, we extracted, for each pair of clusters, the average percentage identity multiplied by the average minimal coverage of receptors from cluster A vs receptors of cluster B and vice versa. We then used the minimum of the two scores (A against B and B against A) as a proxy to describe the distance between the two clusters. The distance matrix was derived from these scores is displayed with a hierarchical clustering. **B) Phylogenies of closely related, alignable clusters.** Since RRNPP systems evolve fast, they are not all alignable in a MSA, and traditional sequence-based approach are therefore not relevant to reconstruct the evolutionary history of all RRNPP systems. However, we provide here local phylogenies of closely-related clusters.
